# Exogenous ANP Treatment Ameliorates Myocardial Insulin Resistance and Protects against Ischemia–Reperfusion Injury in Diet-Induced Obesity

**DOI:** 10.3390/ijms23158373

**Published:** 2022-07-29

**Authors:** Yuhei Oi, Tomohisa Nagoshi, Haruka Kimura, Yoshiro Tanaka, Akira Yoshii, Rei Yasutake, Hirotake Takahashi, Yusuke Kashiwagi, Toshikazu D. Tanaka, Toshiaki Tachibana, Michihiro Yoshimura

**Affiliations:** 1 Division of Cardiology, Department of Internal Medicine, The Jikei University School of Medicine, 3-25-8 Nishi-Shimbashi, Tokyo 105-8461, Japan; yoi@jikei.ac.jp (Y.O.); kimuraha@jikei.ac.jp (H.K.); tanakayoshiro@jikei.ac.jp (Y.T.); a.yoshii@jikei.ac.jp (A.Y.); reiy@jikei.ac.jp (R.Y.); h.takahashi@jikei.ac.jp (H.T.); y-kashiwa@jikei.ac.jp (Y.K.); tanakatd@jikei.ac.jp (T.D.T.); m.yoshimura@jikei.ac.jp (M.Y.); 2 Core Research Facilities for Basic Science, Research Center for Medical Sciences, The Jikei University School of Medicine, 3-25-8 Nishi-Shimbashi, Tokyo 105-8461, Japan; t-tachibana@jikei.ac.jp

**Keywords:** natriuretic peptide, diet-induced obesity, insulin resistance, ischemia–reperfusion injury, intramyocardial lipid droplet

## Abstract

Increasing evidence suggests natriuretic peptides (NPs) coordinate interorgan metabolic crosstalk. We recently reported exogenous ANP treatment ameliorated systemic insulin resistance by inducing adipose tissue browning and attenuating hepatic steatosis in diet-induced obesity (DIO). We herein investigated whether ANP treatment also ameliorates myocardial insulin resistance, leading to cardioprotection during ischemia–reperfusion injury (IRI) in DIO. Mice fed a high-fat diet (HFD) or normal-fat diet for 13 weeks were treated with or without ANP infusion subcutaneously for another 3 weeks. Left ventricular BNP expression was substantially reduced in HFD hearts. Intraperitoneal-insulin-administration-induced Akt phosphorylation was impaired in HFD hearts, which was restored by ANP treatment, suggesting that ANP treatment ameliorated myocardial insulin resistance. After ischemia–reperfusion using the Langendorff model, HFD impaired cardiac functional recovery with a corresponding increased infarct size. However, ANP treatment improved functional recovery and reduced injury while restoring impaired IRI-induced Akt phosphorylation in HFD hearts. Myocardial ultrastructural analyses showed increased peri-mitochondrial lipid droplets with concomitantly decreased ATGL and HSL phosphorylation levels in ANP-treated HFD, suggesting that ANP protects mitochondria from lipid overload by trapping lipids. Accordingly, ANP treatment attenuated mitochondria cristae disruption after IRI in HFD hearts. In summary, exogenous ANP treatment ameliorates myocardial insulin resistance and protects against IRI associated with mitochondrial ultrastructure modifications in DIO. Replenishing biologically active NPs substantially affects HFD hearts in which endogenous NP production is impaired.

## 1. Introduction

Natriuretic peptides (NPs) are hormones produced in the heart that regulate blood pressure and fluid homeostasis through vasodilatory and natriuretic actions and improve cardiac remodeling [1,2,3,4,5,6]. In addition to these classical actions of hemodynamic regulation on the renal and cardiovascular systems, increasing evidence suggests that NPs also regulate the energy balance and glucose homeostasis as well as thermogenesis through interorgan metabolic crosstalk between the heart and other metabolic organ systems, such as adipose tissues [7,8,9,10,11,12,13]. Binding of NPs to NP receptor A (NPR-A) on adipocytes induces triglyceride lipolysis and promotes the uncoupling of mitochondrial respiration by inducing adipose tissue browning, which ameliorates insulin resistance while also activating the thermogenic program [6,14,15,16,17].

Our previous in vitro study demonstrated that in cultured brown adipocytes, A-type NPs (ANPs) raise the intracellular temperature in a low-temperature-sensitive manner via the activation of the p38-UCP1 pathway [18]. In line with this basic research, our clinical study using the cardiac catheter database showed that a decrease in left ventricular ejection fraction (LVEF) is associated with a body temperature decrease, whereas a high plasma B-type NP (BNP) level is associated with a body temperature increase [19]. Another clinical study based on the catheter database indicated that an increase in plasma BNP levels improves insulin resistance and promotes glucose utilization, particularly in patients with acute coronary syndrome, in which glucose is the preferred substrate for cardiac energy metabolism [20,21]. These results are supported by our most recent in vivo study using a high-fat diet (HFD)-induced obese mouse model [6]: Continuous subcutaneous treatment with ANP significantly ameliorated the HFD-induced systemic insulin resistance by attenuating hepatic steatosis and by inducing adipose tissue browning in association with the activation of the brown fat thermogenic program, leading to in vivo thermogenesis during cold exposure. The remarkable findings in the study were that the systemic administration of exogenous ANP has a substantial impact on the morphology and the features of the various adipose tissues and hepatic tissue, leading to a reduction in systemic insulin resistance induced by HFD. However, the impact of exogenous ANP administration on the heart, in which endogenous NPs are abundantly synthesized and secreted, has not been investigated in this particular model.

Acute activation of the serine-threonine kinase Akt, a major downstream target of insulin, is cardioprotective and reduces both infarction and dysfunction after ischemia–reperfusion injury (IRI) [22,23,24]. The increased phosphorylation and activation of Akt in response to IRI or insulin stimulation play an important role in promoting the survival of cardiomyocytes, while a blunted response, such as that seen in diabetic/obese conditions (namely, an insulin-resistant condition), contributes to cardiac dysfunction and IRI aggravation [25,26,27]. Several reports have indicated that NPs also activate Akt signaling; NPs enhance the glucose uptake in human adipocytes through the activation of the NPR-A-cyclic GMP (cGMP)–PKG-Akt-dependent pathway [28], while the activation of the NPR-A-cGMP pathway in skeletal muscle reduces lipotoxicity and improves the sensitivity of insulin-Akt signaling in obese/diabetic-model mice [8]. In the heart, ANP promotes the survival of cardiomyocytes via the cGMP-dependent nuclear accumulation of Akt [29], while direct BNP administration at early reperfusion reduces myocardial injury via the Akt-dependent pathway [30].

Given these previous findings, we hypothesized that exogenous ANP treatment in vivo would ameliorate myocardial insulin resistance and exert cardioprotective effects during IRI, particularly in diet-induced obesity (DIO). To better understand the direct impact of NPs on cardiac tissues in an in vivo DIO model and to determine their functional significance, we used DIO mice with continuous subcutaneous ANP treatment to investigate whether or not exogenous ANP treatment ameliorates insulin resistance in cardiac tissues, similar to observations in adipose and hepatic tissues [6], leading to cardioprotection during IRI through the restoration of Akt signaling.

## 2. Results

### 2.1. Heart Weight and Cardiac Function in HFD Mice

**Figure 1:**
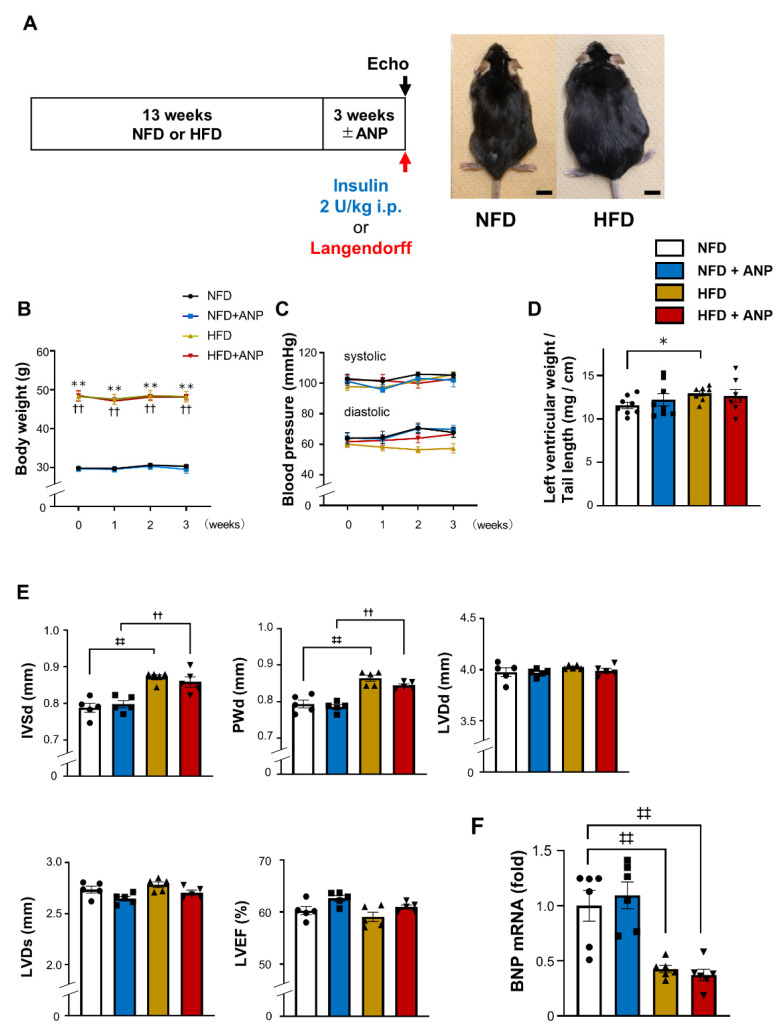
Characteristics of HFD model after treatment with or without ANP. (**A**) A schematic diagram of the experimental protocol and appearance of the obese mice after 13 weeks of HFD feeding. Bars = 1 cm. (**B**) Body weight changes during ANP treatment (n = 6 each). (**C**) Blood pressure changes during ANP treatment (n = 4 each). (**D**) Heart weight at three weeks after treatment with or without ANP (n = 8 each, except for HFD+ANP (n = 7)). (**E**) Echocardiographic parameters at three weeks after treatment with or without ANP (n = 5 each). (**F**) The relative mRNA expression of BNP in left ventricle at three weeks after treatment with or without ANP (n = 6 each). The qPCR data were normalized to glyceraldehyde-3-phosphate dehydrogenase (GAPDH). The data are shown as the fold change normalized to the levels found in the NFD group. * *p* < 0.05 and ** *p* < 0.01 versus NFD, ‡‡ *p* < 0.01 versus NFD, †† *p* < 0.01 versus NFD + ANP. HFD, high-fat diet; IVSd, end-diastolic thickness of intraventricular septum; LVDd, left ventricular end-diastolic dimension; LVDs, left ventricular end-systolic dimension; LVEF, left ventricular ejection fraction; NFD, normal-fat diet; PWd, end-diastolic thickness of posterior wall.

The study design is shown in Figure 1A. After 13 weeks of HFD feeding, mice developed marked obesity with a significant increase in body weight compared with normal-fat-diet (NFD) mice (47.4 ± 0.7 g versus 28.4 ± 0.5 g, *p* < 0.01, Figure 1A,B) consistent with our previous study [6]. We reported that 13-week HFD feeding of this protocol induced systemic glucose intolerance and insulin resistance, which were ameliorated by ANP treatment [6]. ANP treatment for three weeks did not significantly affect the body weight change in either NFD or HFD mice (Figure 1B). Likewise, blood pressure was not changed by either HFD feeding or treatment with ANP during the experimental protocols in the current model (Figure 1C). The heart weight was slightly but significantly increased in HFD mice compared with NFD mice (12.92 ± 0.28 mg/cm versus 11.55 ± 0.37 mg/cm, *p* < 0.05, Figure 1D). This was associated with a significant increase in the left ventricular wall thickness in HFD hearts compared with NFD hearts (Figure 1E). Neither the left ventricular weight nor the wall thickness was affected by ANP treatment. Furthermore, the left ventricular chamber diameters and cardiac function were comparable between HFD and NFD mice, regardless of ANP treatment (Figure 1E). Of note, the gene expression in the left ventricular tissue BNP (NP synthesized primarily in cardiac ventricles) was substantially reduced in HFD hearts compared to NFD hearts (Figure 1F), although exogenous ANP treatment did not significantly affect it, as expected.

### 2.2. ANP Treatment Ameliorated Cardiac Insulin Signaling in HFD Mice

**Figure 2:**
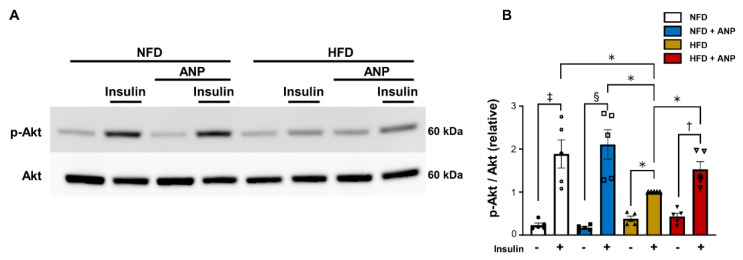
Exogenous ANP treatment ameliorated myocardial insulin resistance. In vivo myocardial insulin sensitivity was assessed by measuring insulin-induced phosphorylation of Akt. (**A**) Representative immunoblots for phospho- and total Akt from cardiac lysates. (**B**) Densitometric quantitation normalized to the level of p-Akt/total Akt expression in HFD with insulin hearts is shown (n = 5 each). * *p* < 0.05 versus HFD, † *p* < 0.05 versus HFD + ANP, ‡ *p* < 0.05 versus NFD, § *p* < 0.05 versus NFD+ANP.

We recently reported that exogenous ANP administration ameliorates HFD-induced insulin resistance by attenuating hepatic steatosis and by inducing adipose tissue browning [6]. We next investigated whether or not exogenous ANP treatment improves myocardial insulin resistance in HFD mice. Phosphorylation of Akt, a major downstream effector of insulin signaling, in response to insulin administration was impaired in HFD hearts, indicating that the HFD mice in the present study manifested cardiac tissue insulin resistance. The blunted response of Akt phosphorylation to insulin stimulation observed in HFD hearts was restored by ANP treatment (Figure 2). These data suggest that exogenous ANP treatment ameliorates myocardial insulin resistance in HFD mice, despite the fact that endogenous NPs are synthesized and secreted in the heart.

### 2.3. Effects of ANP Treatment on the Cardiac Function during IRI in HFD Mice

**Figure 3:**
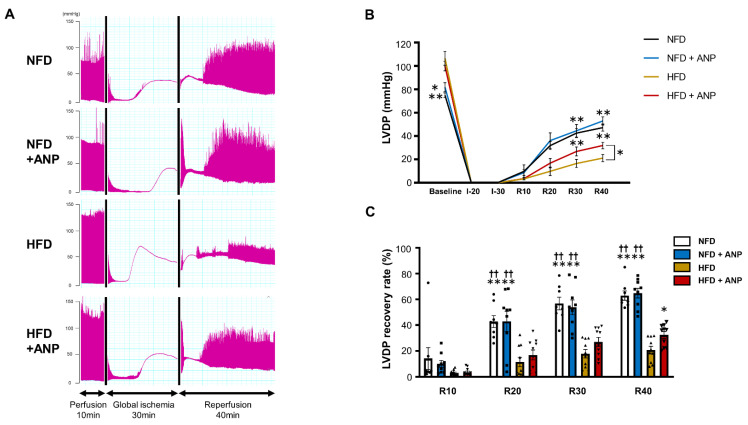
Cardiac functional recovery after IRI with or without ANP treatment. (**A**) Representative tracing of LVDP during ischemia–reperfusion with or without ANP treatment. LVDP profiles (**B**) and LVDP recovery (percent of baseline) (**C**) measured at the indicated time points during ischemia–reperfusion in NFD (n = 8), NFD+ANP (n = 9), HFD (n = 10), and HFD+ANP (n = 10) hearts are shown. * *p* < 0.05 and ** *p* < 0.01 versus HFD, †† *p* < 0.01 versus HFD + ANP. I, ischemia; LVDP, left ventricular-developed pressure; R, reperfusion.

To test the functional significance of the findings that exogenous ANP treatment ameliorated myocardial insulin resistance in HFD mice, hearts were exposed to IRI using the Langendorff model. The representative tracing of left ventricular-developed pressure (LVDP) during IRI is shown in Figure 3A. The baseline cardiac function measured at the end of the 10 min pre-ischemia perfusion period indicated that HFD mice had increased contractile activity, consistent with the previous studies (Table 1) [31,32]. ANP treatment did not significantly affect the baseline cardiac function in the hearts of either NFD or HFD mice. After 30 min of global ischemia followed by 40 min of reperfusion, HFD per se substantially reduced LVDP recovery compared with those values in NFD hearts (20.6 ± 3.0% versus 62.9 ± 3.6% recovery from baseline, *p* < 0.01, Figure 3B,C). Of note, ANP treatment significantly improved cardiac functional recovery in HFD mice (32.3 ± 2.7%, *p* < 0.05 versus HFD without ANP), although ANP did not affect LVDP recovery in NFD (64.8 ± 3.7%). Ischemic contracture, recorded as an increase in diastolic pressure above baseline followed by a continuous rise in pressure after global ischemia, is thought to be initiated by a decrease in the cardiac tissue ATP content [33]. The onset of contracture was defined as a 5 mmHg sigmoid increase in the end-diastolic pressure, and the time from the start of global ischemia to the onset of contracture was assessed (Figure S1). We found that ischemic contracture appeared significantly earlier in HFD hearts than in NFD hearts, suggesting that a decrease in the cardiac tissue ATP during IRI was facilitated in HFD hearts. Although there was no significant time difference in the onset of ischemic contracture in NFD hearts with or without ANP treatment, ANP treatment tended to delay the onset of ischemic contracture in HFD hearts (*p* = 0.07), suggesting that ANP treatment tends to preserve energy in HFD hearts. 

### 2.4. Effects of ANP Treatment on Cardiac Injury after Ischemia–Reperfusion

**Figure 4:**
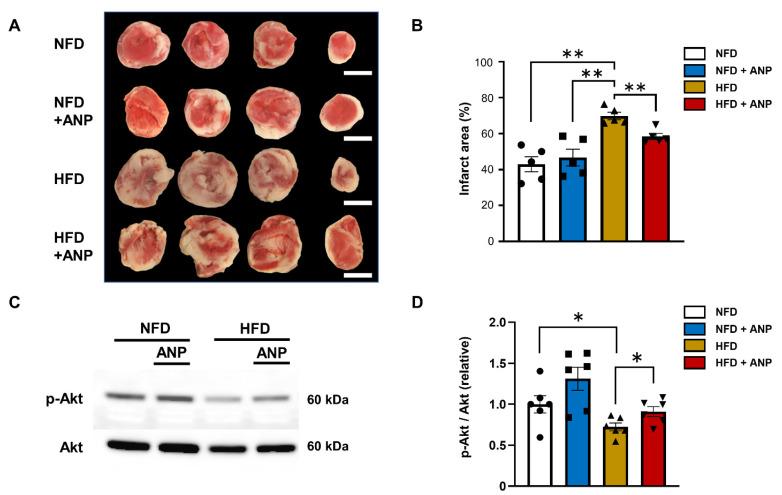
ANP treatment improved IRI and insulin sensitivity in HFD hearts. (**A**) Micrograph showing representative triphenyltetrazolium chloride (TTC) staining of cardiac sections obtained from NFD, NFD + ANP, HFD, and HFD + ANP groups after ischemia–reperfusion. Bars = 3 mm. (**B**) Effects on the quantitated cumulative infarct area size in NFD, NFD + ANP, HFD, and HFD + ANP hearts (n = 5 each). (**C**) Representative immunoblots for phospho- and total Akt from cardiac lysates after ischemia–reperfusion. (**D**) Densitometric quantitation normalized to the level of p-Akt/total Akt expression in NFD hearts after IRI is shown (n = 6 each). * *p* < 0.05 and ** *p* < 0.01 versus HFD.

Consistent with the findings of the cardiac functional recovery after ischemia–reperfusion, significantly larger infarcts were observed in HFD- compared to NFD-fed mice regardless of ANP, and these findings were reduced by exogenous ANP administration (43.0 ± 4.2% (NFD), 46.7 ± 4.67% (NFD+ANP), and 58.4 ± 1.7% (HFD+ANP) versus 69.9 ± 2.0% (HFD), *p* < 0.01, n = 5 each, Figure 4A,B). These data suggest that exogenous ANP treatment improves cardiac functional recovery and reduces myocardial injury after ischemia–reperfusion, particularly in the hearts of mice with DIO.

### 2.5. Effects of ANP Treatment on Insulin Signaling during IRI

To explore the mechanisms underlying the cardioprotective effects of ANP treatment in HFD hearts during IRI, we examined the activity of the insulin signaling pathway. Phosphorylation of Akt in response to IRI was impaired in HFD hearts, which was restored by ANP treatment (Figure 4C,D). These data suggest that exogenous ANP treatment ameliorates myocardial insulin resistance during IRI in HFD mice.

### 2.6. Effects of ANP Treatment on Myocardial Ultrastructure

**Figure 5:**
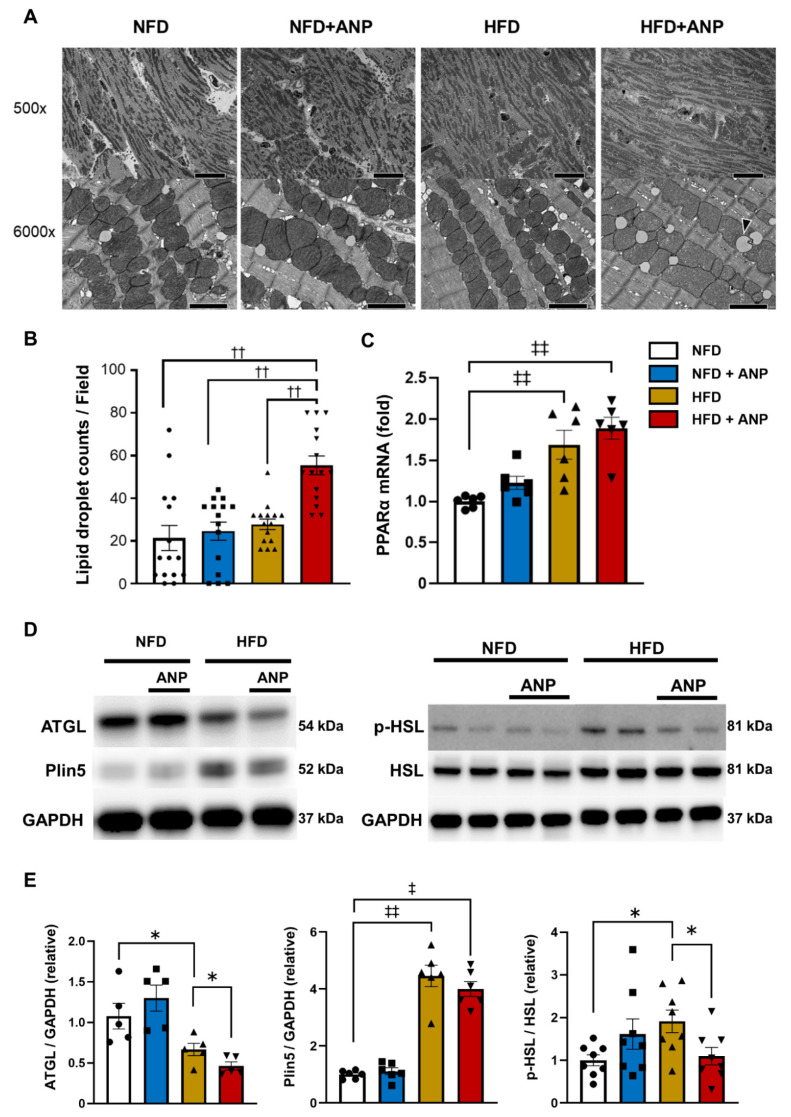
Myocardial microstructure and LD regulatory protein expression in vivo. (**A**) Representative electron micrographs of heart sections before IRI with or without ANP. Each pair of electron micrographs displayed a set of lower magnification (top; scale bar, 2.0 μm) and higher magnification (bottom; scale bar, 20.0 μm). The arrow heads indicate LD. (**B**) Quantification of LD content before IRI with or without ANP treatment is shown (n = 3 each). (**C**) The relative mRNA expression of PPARα in left ventricle (n = 6 each). The qPCR data were normalized to GAPDH. The data are shown as the fold change normalized to the levels found in the NFD group. (**D**) Representative immunoblots for ATGL, Plin5, phospho-, and total HSL from non-ischemic cardiac lysates. (**E**) Densitometric quantitation normalized to the level of each protein expression in NFD hearts is shown (ATGL n = 5 each, Plin5 n = 6, HSL n = 8 each). GAPDH immunoblotting is shown as a loading control. ‡ *p* < 0.05 and ‡‡ *p* < 0.01 versus NFD, * *p* < 0.05 versus HFD, †† *p* < 0.01 versus HFD + ANP. ATGL, adipose triglyceride lipase; GAPDH, glyceraldehyde 3-phosphate dehydrogenase; HSL, hormone-sensitive lipase; Plin5, perilipin 5; PPARα, peroxisome-proliferator-activated receptor alpha.

To further determine the effects of exogenous ANP administration on myocardial ultrastructural changes, we evaluated the mitochondrial morphology and lipid droplet (LD) distribution using electron microscopy (Figure 5A and Figure S2A). Before ischemia, there were no significant between-group differences in the structure of mitochondria, and relatively few damaged mitochondria were seen, even in HFD hearts (Figure 5A). In contrast, the number of LDs, most of which resided adjacent to mitochondria, was significantly increased in HFD mice having undergone ANP treatment (Figure 5A,B and Figure S2A), indicating that ANP protects mitochondria from lipid overload by trapping lipids [34,35,36]. The mRNA levels of peroxisome-proliferator-activated receptor α (PPARα), the principal transcriptional regulator of fatty acid oxidation enzyme genes [37], were significantly increased in HFD hearts, regardless of ANP treatment (Figure 5C), which may reflect the compensatory response to the facilitated fatty acid uptake into the myocardium [38]. To explore the mechanism by which myocardial LDs were increased in HFD mice which had undergone ANP treatment, we examined the expression of cardiac perilipin 5 (Plin5) (which is abundantly expressed in the heart [27,34]), the anchored proteins on LD surfaces regulating LD generation/formation (namely, protecting LDs from lipase attack) [34], and adipose triglyceride lipase (ATGL) and hormone-sensitive lipase (HSL), major lipases that facilitate lipolysis in the cardiac tissue, thus leading to a decrease in the number of LDs [34,39,40]. Consistent with the previous findings that Plin5 expression is positively regulated by PPARα [34], Plin5 expression was significantly increased in HFD hearts, regardless of ANP treatment (Figure 5D,E), although its function (i.e., activity) remains to be determined. The ATGL expression was significantly decreased in HFD hearts (Figure 5D,E), consistent with the previous study [41]. Of note, ATGL levels were further decreased in HFD hearts with ANP treatment (Figure 5D,E). In contrast, the levels of p-HSL, the active form of HSL that induces lipolysis, were significantly increased in HFD hearts, again presumably reflecting the compensatory response. We can also infer that increased p-HSL hydrolyzes various lipid esters, leading to lipid overload of the surrounding subcellular organelles, which ultimately results in increased IRI [42]. This may also explain the finding that the number of LDs was not significantly increased in HFD hearts compared to NFD hearts. However, it should be noted that ANP treatment substantially decreased p-HSL in HFD hearts, in parallel with the results of ATGL expression (Figure 5D,E). These data are in line with the electron microscopic findings showing that the number of LDs was increased in HFD hearts having undergone ANP treatment (Figure 5A and Figure S2A).

### 2.7. Effects of ANP Treatment on Mitochondrial Damage after IRI

**Figure 6:**
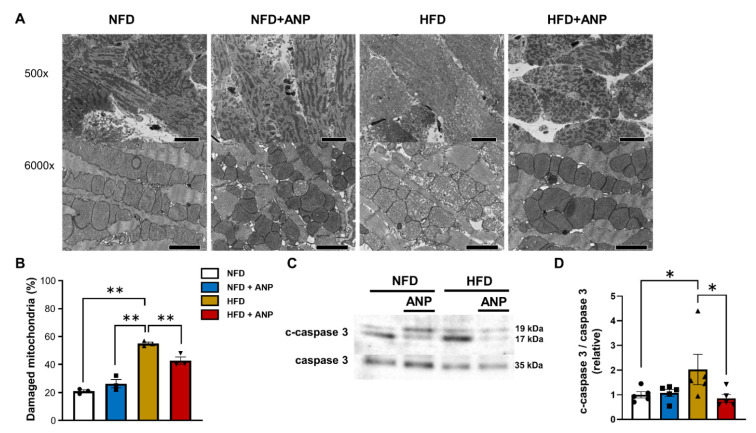
ANP treatment alleviated mitochondrial damage after IRI in HFD hearts. (**A**) Representative electron micrographs of heart sections after IRI with or without ANP. Each pair of electron micrographs displayed a set at lower magnification (top; scale bar, 2.0 μm) and higher magnification (bottom; scale bar, 20.0 μm). (**B**) Number of damaged mitochondria after IRI. (**C**) Representative immunoblots for cleaved and total caspase 3 from cardiac lysates after IRI. (**D**) Densitometric quantitation normalized to the level of cleaved/total caspase 3 expression in NFD hearts after IRI is shown (n = 5 each). * *p* < 0.05 and ** *p* < 0.01 versus HFD.

After ischemia–reperfusion, the number of damaged and swollen mitochondria presenting with cristae disruption was dramatically increased in HFD hearts compared to NFD hearts. However, exogenous ANP treatment substantially attenuated mitochondria cristae disruption in HFD hearts (Figure 6A,B and Figure S2B). Consistent with the electron microscopy findings, cleaved caspase 3 levels, a major indicator of apoptosis, were significantly increased in HFD hearts after IRI, which was substantially alleviated by ANP treatment (Figure 6C,D).

## 3. Discussion

**Figure 7:**
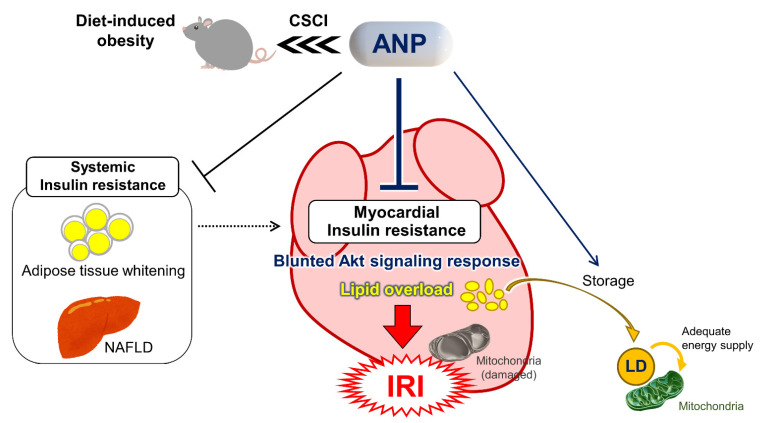
Exogenous ANP treatment in diet-induced obesity ameliorates not only systemic insulin resistance but also myocardial insulin resistance and protects against ischemia-reperfusion injury, which is associated with significant modifications of mitochondrial ultrastructure. CSCI, continuous subcutaneous infusion; IRI, ischemia-reperfusion injury; LD, lipid droplet; NAFLD, non-alcoholic fatty liver disease.

In the present study, we found that exogenous ANP administration significantly ameliorated HFD-induced myocardial insulin resistance, as indicated by the restoration of the insulin-Akt signaling pathway. Accordingly, the continuous subcutaneous ANP treatment in HFD mice improved the cardiac functional recovery and reduced myocardial injury after IRI along with the restoration of the disrupted mitochondrial ultrastructure in HFD hearts (Figure 7). The remarkable findings in the present study are that the systemic administration of exogenous ANP had a substantial impact on the ultrastructural morphology, the molecular signaling pathway involved in energy metabolism, and the function of the failing myocardium—the tissue in which a vast number of endogenous NPs are produced and secreted.

Using the exact same model of DIO mice having undergone continuous subcutaneous ANP treatment [6], we recently reported that exogenous ANP treatment ameliorates systemic insulin resistance by attenuating hepatic steatosis and inducing adipose tissue browning in association with the activation of the brown fat thermogenic program. In the present study, we found that exogenous ANP treatment also significantly ameliorated the local tissue insulin resistance in the hearts of HFD mice, although it had little influence on the hearts of NFD mice. Thus, it is possible that the present findings may be secondary to improvement in systemic insulin resistance (Figure 7) [6]. These results are consistent with those of a previous study showing that chronic BNP treatment improves the metabolic profile and prevents the development of myocardial dysfunction in diabetic mice [16], although those authors did not investigate its effects on IRI or the myocardial ultrastructure. However, that previous study and our present study share a common finding that NPs play a cardioprotective role that is particularly evident in obese diabetic subjects. Unexpectedly low NP levels in proportion to the heart failure severity are often observed in obese/diabetic subjects, although the mechanisms have yet to be fully established [43,44]. The proposed mechanisms involve not only increasing NP clearance due to a high expression of NP clearance receptor [8,12,14,17,28] and/or higher activities of neprilysin [11,45], but also an impaired production of “biologically active” NPs [46,47,48]. Therefore, replenishment of biologically active NPs might be more effective in subjects with DIO than in normal lean subjects, in whom endogenous cardiac NP production is substantially reduced, as shown in Figure 1F. There is a question of why elevated endogenous “circulating” ANP in DIO mice showed little impact in the present study (See Materials and Methods). One can speculate that the “cardiac tissue” levels of endogenous NPs as well as their bioactivity are still too insufficient to have a substantial impact on the heart per se in DIO mice, given the findings described above. However, it is also possible that it might be related to a modification in the affinity of ANP for its specific receptor or in a downstream signal transduction in HFD hearts, thus requiring a higher concentration of ANP. Meanwhile, other previous studies using the ex vivo Langendorff ischemia–reperfusion model showed the direct protective effects of BNP perfusion on the non-diabetic heart [30,49]. However, given that we did not observe any significant impact of exogenous ANP on NFD hearts in the present study, the effects of systemic continuous treatment with exogenous NPs (in contrast to direct short-period perfusion) on cardioprotection against IRI might be deeply involved in the “insulin resistance” of both systemic and local cardiac tissues observed in DIO mice. Taken together, the findings in the present study that the impact of exogenous ANP treatment is particularly evident in DIO subjects can be explained by the replenishment of biologically active NPs in HFD hearts in which endogenous NP production is impaired as well as by the restoration of decreased cardiac tissue insulin sensitivity in DIO mice. However, conversely, exogenous ANP treatment might have a limited impact on NFD hearts in which neither endogenous NP production nor insulin/stress-induced Akt signaling activation (superinduction) is impaired.

Cardiac dysfunction and myocardial injury observed in HFD-induced obesity are often associated with various molecular pathologies, represented by disturbed insulin-Akt signaling activation, namely myocardial insulin resistance [25,26]. Insulin-Akt signaling is a key regulator of cardiomyocyte survival and function as well as an important modulator of metabolic substrate utilization [21,22,50]. It is widely accepted that acute activation—or, to be more precise, acute acceleration (superinduction)—of insulin-Akt signaling during IRI has cardioprotective effects both in vitro and in vivo [21,22,23,50,51] through its direct anti-apoptotic effects as well as by promoting glucose utilization, which is critical for the metabolism of the failing myocardium, such as during IRI [21,52]. Akt can also be directly activated by NPs in various tissues [8,28], including the heart [29,30]. However, it is also possible that NP administration might inhibit the production of adipokines linked to inflammation and insulin resistance [53], which would result in the promotion of myocardial insulin sensitivity, leading to the enhanced Akt signaling activation in response to insulin stimulation as well as IRI. Thus, it is likely that continuous ANP administration in the present study restored Akt signaling in HFD hearts through either the direct action of NPs on the heart or the indirect action of improving myocardial insulin sensitivity via interorgan metabolic crosstalk with adipose tissues. Further studies are warranted to fully delineate the precise mechanism by which NPs activate Akt signaling, which is particularly evident in the HFD heart.

Intramyocardial LDs are dynamic organelles with diverse properties and functions that play a critical role in maintaining lipid metabolism homeostasis [27,34,35]. Several previous studies have shown that the tissue contents of triglyceride and/or lipotoxic ceramides as well as other fatty acid metabolites are increased in obese diabetic hearts [38,54,55,56], although what kinds of lipids (esters) or their metabolites are actually accumulated in the cardiac tissue of the current HFD model remains uncertain. Excess fatty acids consumed by cardiomyocytes are converted into triglycerides and stored (sequestered) in LDs, thus protecting the myocardium from lipotoxicity by reducing lipid overload on mitochondria [27,34,35,36]. Although the LD content in HFD hearts in the present study was comparable to that in NFD hearts, similar to a previous study by others [57], we noted marked numbers of LDs in HFD hearts treated with ANP. One can infer that exogenous ANP in HFD hearts may prevent excessive lipolysis and protect the mitochondria adjacent to LDs from lipid-overload-induced oxidative stress by preserving LD and storing excess fatty acids (Figure 7). As such, these effects of ANP are only particularly evident in DIO subjects in which there is an excess fatty acid overload, with little influence seen in NFD subjects. In support of this idea, similar findings were reported previously, where empagliflozin, a selective SGLT2 inhibitor, alleviated mitochondrial damage after myocardial infarction in diabetic rat hearts along with LD accumulation [36].

While previous studies reported that NPs induce (rather than prevent) lipolysis in adipose tissues [6,14,15,16,17], the regulation of LD formation may differ between cardiac and non-cardiac tissues [39]. In fact, we found that ANP treatment decreased the ATGL expression in HFD hearts and suppressed HFD-induced HSL phosphorylation, which might account for the myocardial LD preservation. In agreement with this, a previous study showed that Plin5 inhibited the lipolysis of LD and alleviated myocardial IRI by reducing oxidative stress along with Akt signaling activation [27]. Thus, further studies are warranted to fully delineate the mechanisms underlying the direct regulations of ANP on these lipases as well as the Plin5 function.

In contrast to the myocardial ultrastructure at baseline before IRI, damaged mitochondria with sparse cristae were more evident in HFD hearts than in NFD hearts after IRI, and exogenous ANP substantially alleviated the mitochondrial damage in HFD mice. This was consistent with the data showing that ANP treatment suppressed IRI-induced cleaved caspase 3 levels (Figure 6C,D). We previously reported, using in vitro-cultured cardiomyocytes, that exogenous ANP treatment has a direct antioxidant effect on the failing heart, which might be at least partly due to mitochondrial protection [58]. The current two major findings—impaired Akt signaling and mitochondrial damage—observed in the HFD myocardium after ischemia–reperfusion may be the chicken or the egg (or may have synergistic influence); exogenous ANP may have alleviated the mitochondrial damage and improved the mitochondrial function through the proposed mechanisms described above (e.g., anti-oxidant, anti-lipotoxic effects), leading to a decrease in myocardial insulin resistance and an increase in insulin sensitivity; alternatively, exogenous ANP may have activated Akt signaling in the HFD hearts, leading to the alleviation of mitochondrial damage, presumably through anti-apoptotic effects and/or regulation of the mitochondrial permeability transition pore [51].

Several limitations associated with the present study warrant mention. The “classical actions” of hemodynamic regulation by NPs on the renal and cardiovascular systems may have had some impact on the cardiac functional recovery after IRI, although various hemodynamic parameters at baseline (before IRI), such as the blood pressure (Figure 1C), serum creatinine levels [6], and echocardiographic parameters (Figure 1E), were not significantly affected by ANP treatment in the present model. In vivo studies of IRI would help clarify the effects of the systemic interorgan network on cardiac function and myocardial injury during the ischemia–reperfusion period, including systemic influences from vascular tone, neurohumoral input, systemic substrate metabolism, and the presence of circulating hematopoietic cells. However, the Langendorff system remains useful for avoiding such systemic confounders and better understanding the intrinsic mechanisms controlling myocardial insulin resistance and the cardiomyocyte survival after IRI. Furthermore, although glucose was the only substrate used in the present ex vivo Langendorff perfusion model, which does not completely mimic physiological substrates in vivo, it remains an important preferential substrate for cardiac energy metabolism under pathological conditions, such as the acute phase of IRI [21,59]. Thus, we believe that the IRI data in the present study still reflect in vivo conditions to some extent, even when using the current Langendorff system. However, the present study would be strengthened if the cardioprotective effects of ANP during IRI could be reflected under more physiological conditions, such as either in vivo or ex vivo isolated hearts perfused with fatty acid-containing buffer. Other limitations associated with the present study are the same as those described in our recent study [6]. For example, the expression of NPR-C in rodents is reported to be approximately 100-fold higher than that in humans [15,18]. In the present study, however, the experiments were performed under basically fasting conditions, during which the NPR-A expression was upregulated, while the NPR-C expression was downregulated [14,60]. In addition, exogenous ANP was administered at a pharmacological dose, although it did not affect either the blood pressure profile or body weight. Therefore, our findings suggest that exogenous ANP may still have a significant influence, even in the rodent model used in the present study [6].

## 4. Materials and Methods

### 4.1. Animal Models

All animal procedures conformed to the National Institutes of Health Guide for the Care and Use of Laboratory Animals and were approved by the Animal Research Committee at the Jikei University School of Medicine (2016-038C8). All animal experiments were carried out in accordance with the ARRIVE guidelines. The study design is shown in Figure 1A. Male C57BL/6 mice at 8 weeks of age were fed either a normal-fat diet (NFD) or HFD for 13 weeks, as previously described [6]. Where indicated, mice fed an NFD or HFD received ANP (0.5 μg/kg/min) (carperitide, kindly provided by Daiichi-Sankyo Pharmaceutical Co., Tokyo, Japan) subcutaneously via a mini-osmotic pump (model 2004; Alzet Corporation, CA, USA) for three weeks, also as previously described [6]. The control group received pumps containing sterile water only. We confirmed that ANP administration by this method substantially increased serum ANP levels in both NFD (65.4 ± 3.4 pg/mL vs. 176.2 ± 23.6 pg/mL, *p* < 0.05) and HFD mice (155.8 ± 10.0 pg/mL vs. 236.6 ± 26.1 pg/mL, *p* < 0.05) [6]. All mice were housed at room temperature (25 °C). Body weight and blood pressure were measured weekly during the study period. The blood pressure in conscious mice was measured using a noninvasive computerized tail-cuff system (BP-98A-L, Softron Co., Ltd., Tokyo, Japan), as previously described [6].

### 4.2. Echocardiography

Echocardiography was performed using a high-resolution Vevo 3100 system (VisualSonics) equipped with a high-frequency ultrasound probe, as previously described [61]. The 2D M-mode was obtained at the level of the papillary muscle, and the end-diastolic thickness of the intraventricular septum (IVSd), left ventricular end-diastolic dimension (LVDd), end-diastolic thickness of the posterior wall (PWd), and left ventricular end systolic dimension (LVDs) were measured. The left ventricular ejection fraction (LVEF) was calculated using the Vevo 3100 software program (VisualSonics). All measurements were obtained in triplicate and averaged.

### 4.3. Insulin Sensitivity Test

Insulin sensitivity was examined 10 min after an intraperitoneal injection of insulin (2 IU /kg) or an equal volume of phosphate-buffered saline (PBS, control). After cervical dislocation, heart tissue samples were isolated and snap-frozen in liquid nitrogen. Protein extracts were immunoblotted for well-known downstream signals of insulin action, i.e., phosphorylated and total Akt [22].

### 4.4. Experiments in Langendorff Hearts

After 12 to 20 h of fasting, mice were heparinized (1000 IU/kg, i.p.) and anesthetized (0.3 mg/kg of medetomidine, 4.0 mg/kg of midazolam, and 5.0 mg/kg of butorphanol, i.p.) in order to eliminate suffering. The heart was then rapidly excised, and the aorta was cannulated onto a Langendorff apparatus, followed by retrograde perfusion at a constant pressure (80 mmHg) with modified Krebs–Henseleit buffer (11 mM glucose, 118 mM NaCl, 4.7 mM KCl, 2.0 mM CaCl_2_, 1.2 mM MgSO_4_, 1.2 mM KH_2_PO_4_, 25 mM NaHCO_3_, 0.5 mM EDTA), as previously described [22,32]. A temperature-regulated heart chamber was then placed around the heart in order to keep the perfused heart at a certain temperature. Cardiac hemodynamics was measured using a water-filled balloon catheter introduced into the left ventricle.

### 4.5. Ischemia–Reperfusion Model

After a stabilization period of 20 min, the control hearts were perfused for another 10 min before ischemia–reperfusion in order to measure the baseline pre-ischemia cardiac function. Subsequently, global ischemia was applied by eliminating flow for 30 min followed by 40 min of reperfusion, as previously described [32]. For the immunoblotting analysis, the individual perfused hearts were snap-frozen in liquid nitrogen and stored at −80 °C prior to protein extraction. Triphenyltetrazolium chloride (TTC) staining was performed to determine the myocardial infarct size using the individual perfused hearts after ischemia–reperfusion, as previously described [22,32]. For each section, the area at risk (AAR) and infarct area were measured from enlarged digital micro-graphs. Percent myocardial infarction (%MI) was calculated as the total infarction area divided by the total AAR for that heart.

### 4.6. Immunoblotting

Immunoblotting was performed as previously described [32,61] with rabbit polyclonal Phospho-Akt (Ser473) antibody (1:1000, #9271; Cell Signaling Technology, Tokyo, Japan), Akt antibody (1:1000, #9272; Cell Signaling Technology, Tokyo, Japan), ATGL antibody (1:1000, #2138; Cell Signaling Technology, Tokyo, Japan), Phospho-HSL (S563) antibody (1:1000, #4139; Cell Signaling Technology, Tokyo, Japan), HSL antibody (1:1000, #4107; Cell Signaling Technology, Tokyo, Japan), Plin5 antibody (1:1000, GP-31; PROGEN Biotechnik, Germany), and GAPDH antibody (1:5000, #2118; Cell Signaling Technology, Tokyo, Japan). The signals were detected using chemiluminescence.

### 4.7. RNA Isolation, Reverse Transcription (RT) and Real-Time Polymerase Chain Reaction (PCR)

Total RNA was extracted from the frozen tissues using TRIzol reagent (Invitrogen), and a quantitative real-time PCR was performed using a StepOnePlus Real-time PCR System and the StepOne Software program (Applied Biosystems), as described previously [6]. The real-time PCR protocol consisted of one cycle at 95 °C for 20 s followed by 40 cycles at 95 °C for 1 s and 60 °C for 20 s using the primers for Nppb (Mm01255770_g1; Applied Biosystems), Ppara (Mm00440939_m1; Applied Biosystems), and Ppargc1 (Mm01208835_m1; Applied Biosystems). The transcriptional levels were determined using the ΔΔCt method with normalization to GAPDH (Mm99999915_g1; Applied Biosystems).

### 4.8. Electronic Microscope

Small pieces of left ventricular myocardium were obtained from the mid-left ventricular wall of mice by immersion in 2% glutaraldehyde in 0.1 M phosphate buffer (pH 7.3) overnight at 4 °C; they were then postfixed in 1% osmium tetroxide in the same buffer for 2 h at 4 °C, dehydrated in ethanol, immersed in absolute propylene oxide, and embedded in Epok 812 (Oken, Tokyo, Japan). Ultrathin sections were cut with a diamond knife and stained with uranyl acetate and lead citrate before being observed with a JEM-1400Plus (JEOL, Tokyo, Japan) electron microscope at 80 kV. A uniform sampling of 25 electron micrographs was utilized for the morphometric assay of each group. Five random fields, micrographed at 500× from each of the 3 blocks, were printed at a final magnification of 6000× and analyzed using ImageJ software for the calculation of the LD number and damaged mitochondrial number.

### 4.9. Statistical Analysis

The data are presented as the mean ± standard error of the mean of at least three independent experiments. Student’s t-test was used for the comparison of two data sets. The echocardiographic data, hemodynamic parameters and %MI determined according to TTC staining were compared using one-way analysis of variance (ANOVA) followed by post hoc Dunnett or Bonferroni tests. Immunoblots and PCR were compared using the Kruskal–Wallis test and Mann–Whitney U test. All statistical calculations were performed using GraphPad Prism 9. A value of *p* < 0.05 was considered to be significant.

## 5. Conclusions

The systemic administration of ANP ameliorated myocardial insulin resistance and protected against IRI, independent of the known systemic hemodynamic effects. This was associated with the alleviation of the disrupted mitochondrial structure in HFD hearts after ischemia–reperfusion. The finding that exogenous NPs have a significant impact on NP-rich failing myocardium provides new insight into the biological properties and dynamics of NPs. 

## Figures and Tables

**Table 1:** Baseline cardiac function of ex vivo perfused hearts with or without ANP treatment. ‡ *p* < 0.05 and ‡‡ *p* < 0.01 versus the NFD group. HFD, high-fat diet; HR, heart rate; LVDP, left ventricular-developed pressure; LVEDP, left ventricular end-diastolic pressure; LVSP, left ventricular systolic pressure; NFD, normal-fat diet; RPP, rate pressure product.

	NFD	NFD+ANP	HFD	HFD+ANP
	(n = 14)	(n = 14)	(n = 13)	(n = 13)
LVSP, mmHg	92.5 ± 3.7	91.4 ± 3.0	121.0 ± 3.7 ‡‡	111.4 ± 3.3 ‡‡
LVEDP, mmHg	8.9 ± 0.7	7.8 ± 0.7	9.6 ± 0.7	9.8 ± 0.7
LVDP, mmHg	83.6 ± 3.5	83.5 ± 2.9	105.8 ± 5.2 ‡‡	101.6 ± 3.5 ‡‡
+dp/dt, mmHg/s	2360 ± 169	2338 ± 125	3214 ± 256 ‡‡	2746 ± 136
−dp/dt, mmHg/s	−2227 ± 192	−2204 ± 153	−2980 ± 285 ‡	−2468 ± 126
HR, bpm	268 ± 17	280 ± 19	302 ± 16	248 ± 18
RPP, mmHg·bpm	25,314 ± 2422	25,981 ± 2454	35,733 ± 2564 ‡	27,690 ± 2200
Coronary flow, mL/min	3.06 ± 0.28	3.30 ± 0.35	4.17 ± 0.31 ‡	3.08 ± 0.31

## Data Availability

The data that support the findings of this study are contained within the article and the Supplementary Material, and also are available from the corresponding author (T.N.) upon reasonable request.

## Supplementary Materials

The supporting information can be downloaded at: https://www.mdpi.com/article/10.3390/ijms23158373/s1.

## Abbreviations

ANP	A-type natriuretic peptide
ATGL	adipose triglyceride lipase
BNP	B-type natriuretic peptide
DIO	diet-induced obesity
GAPDH	glyceraldehyde-3-phosphate dehydrogenase
HFD	high-fat diet
HSL	hormone-sensitive lipase
IRI	ischemia–reperfusion injury
LD	lipid droplet
LVDP	left ventricular-developed pressure
NFD	normal-fat diet
NPR-A	natriuretic peptide receptor A
NPs	natriuretic peptides
Plin5	perilipin 5
PPARα	peroxisome-proliferator-activated receptor alpha
TTC	triphenyltetrazolium chloride
