# Supplementary material for: Exogenous ANP Treatment Ameliorates Myocardial Insulin Resistance and Protects against Ischemia–Reperfusion Injury in Diet-Induced Obesity
Source: Int J Mol Sci. 2022 Jul 29;23(15):8373. doi: 10.3390/ijms23158373 (PMC9369294; doi:10.3390/ijms23158373)
Supplement: Supplementary file 1 [file ijms-23-08373-s001.zip › ijms-1825251-supplementary.pdf]

Supplementary Materials

Exogenous ANP treatment ameliorates myocardial insulin resistance and protects against ischemia-reperfusion injury in diet-induced obesity.

Yuhei Oi,[1] Tomohisa Nagoshi,[1] Haruka Kimura,[1] Yoshiro Tanaka,[1] Akira Yoshii,[1] Rei Yasutake,[1] Hirotake Takahashi,[1] Yusuke Kashiwagi,[1] Toshikazu D. Tanaka,[1] Toshiaki Tachibana,[2] and Michihiro Yoshimura.[1]

[1]Division of Cardiology, Department of Internal Medicine, The Jikei University School of Medicine
[2]Core Research Facilities for Basic Science, Research Center for Medical Sciences, The Jikei University School of Medicine

Correspondence to: Tomohisa Nagoshi, M.D., Ph.D.
3-25-8, Nishi-Shimbashi, Minato-ku, Tokyo, 105-8461, JAPAN
Tel: +81-3-3433-1111 (ex.3261), Fax: +81-3-3459-6043
E-mail: tnagoshi@jikei.ac.jp

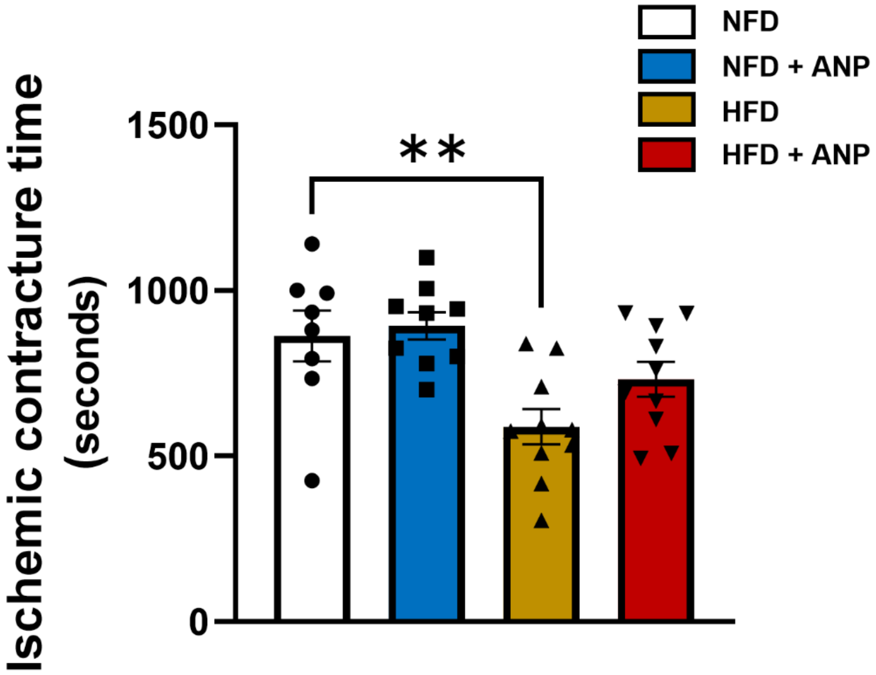

Supplemental Figure S1. Ischemic contracture time.

Ischemic contracture, recorded as an increase in diastolic pressure above baseline followed by a continuous rise in pressure after global ischemia, is thought to be initiated by a decrease in the cardiac tissue ATP content. The onset of contracture was defined as a 5-mmHg sigmoid increase in the end-diastolic pressure, and the time from the start of global ischemia to the onset of contracture was assessed (NFD n=8, NFD+ANP n=9, HFD, HFD+ANP n=10). **P<0.01 versus HFD.

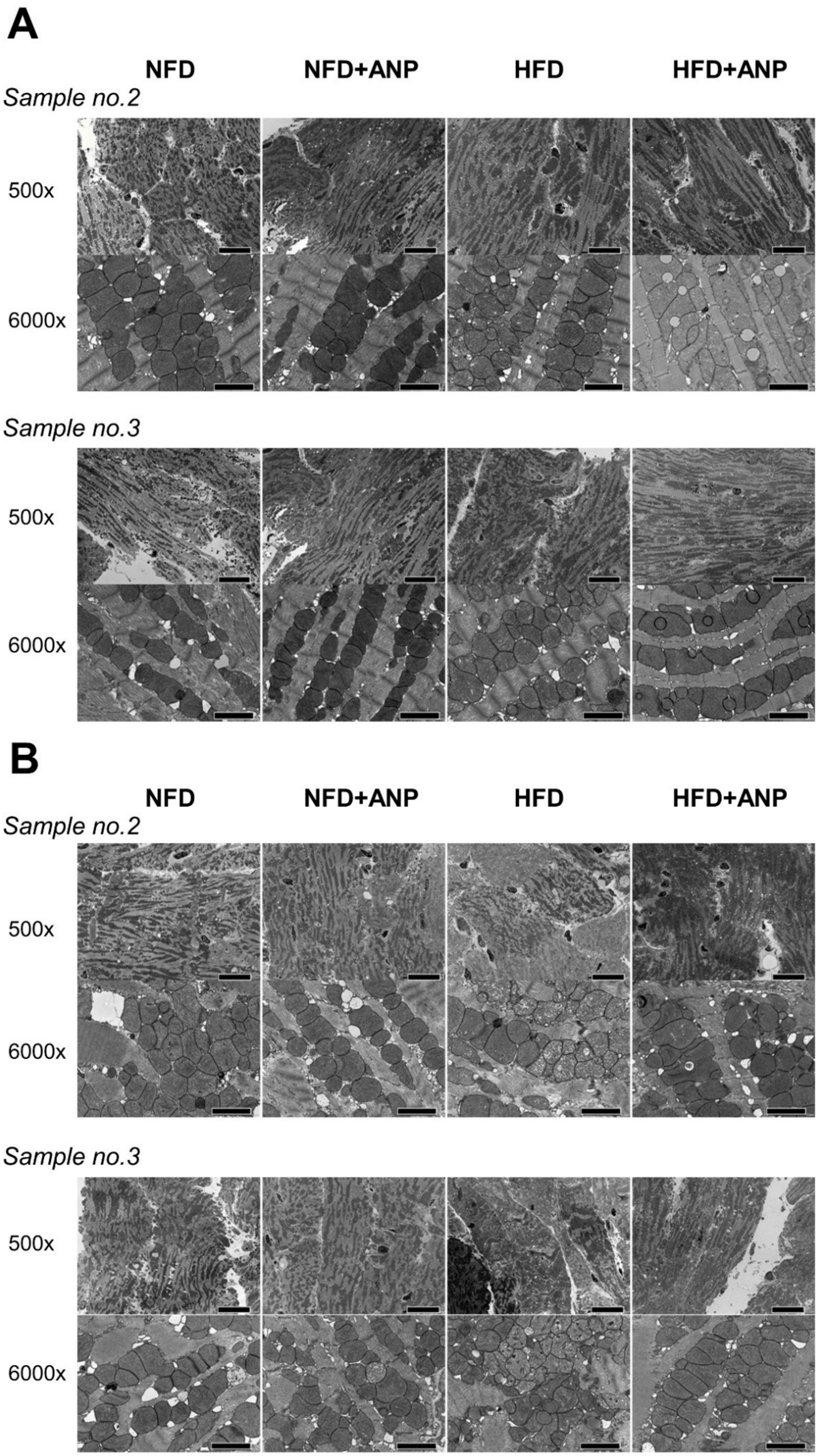
A
NFD
NFD+ANP
HFD
HFD+ANP
Sample no.2
500x
6000x
Sample no.3
500x
6000x
B
NFD
NFD+ANP
HFD
HFD+ANP
Sample no.2
500x
6000x
Sample no.3
500x
6000x

Supplemental Figure S2. Myocardial microstructure before and after ischemia-reperfusion (two remaining samples).

Representative electron micrographs of heart sections before **(A)** and after **(B)** ischemia-reperfusion with or without ANP. Each pair of electron micrographs displayed a set of lower magnification (top; scale bar, 2.0 μm) and higher magnification (bottom; scale bar, 20.0 μm).
